# The effect of high concentration oxygen therapy on PaCO_2_ in acute and chronic respiratory disorders

**DOI:** 10.1186/2213-0802-1-8

**Published:** 2013-04-04

**Authors:** Janine Pilcher, Kyle Perrin, Richard Beasley

**Affiliations:** 1 Medical Research Institute of New Zealand, Private Bag 7902, Wellington, 6242 New Zealand; 2 Wellington Regional Hospital, Capital & Coast District Health Board, Private Bag 7902, Wellington, 6242 New Zealand; 3 School of Biological Sciences, Victoria University of Wellington, PO Box 600, Wellington, 6140 New Zealand

## Abstract

There is evidence that the potential for high concentration oxygen therapy to increase PaCO_2_ is not limited to stable and acute exacerbations of COPD, but also to other acute respiratory disorders with abnormal gas exchange such as asthma and pneumonia, and chronic respiratory conditions with hypercapnia such as obesity hypoventilation syndrome. This evidence forms the basis of consensus guidelines which recommend that oxygen therapy is titrated in COPD and other respiratory conditions, to ensure the maximal benefits of oxygen therapy are achieved while reducing the potential for harm due to hyperoxia.

**Electronic supplementary material:**

The online version of this article (doi:10.1186/2213-0802-1-8) contains supplementary material, which is available to authorized users.

## Introduction

The risks associated with high concentration oxygen therapy in acute exacerbations of chronic obstructive pulmonary disease (COPD) were reported over 50 years ago [1]. Since then it has been demonstrated that high concentration oxygen therapy can cause an increase in PaCO_2_ in both stable COPD and exacerbations of COPD, and that in some patients the effect can be both rapid and marked with an increase in PaCO_2_ of >20 mmHg within 60 minutes [2]. The clinical significance of this effect is evident from the recent randomised controlled trial (RCT) which demonstrated that in acute exacerbations of COPD, high concentration oxygen therapy in the pre-hospital setting significantly increases mortality compared with a titrated regimen to achieve arterial oxygen saturations between 88% and 92% [3]. In patients with confirmed COPD who received oxygen therapy treatments as per protocol, the PaCO_2_ was 34 mmHg higher in the high concentration oxygen therapy group (Table 1).

**Table 1:** **High concentration versus titrated oxygen therapy in the pre-hospital setting in patients with confirmed COPD**

	High concentration	Titrated	Relative risk (95% CI)	Difference	P Value
Mortality	9%	2%	0.22 (0.5 to 0.91)		0.04
Ventilation	14%	10%	0.67 (0.29 to 1.54)		0.34
Arterial blood gases†					
Mean (SD) pH	7.29 (0.15)	7.41 (0.09)		0.12	0.01
Mean (SD) PaCO_2_ (mmHg)	76.5 (50.2)	42.9 (14.2)		−33.6	0.02
Mean (SD) PaO_2_ (mmHg)	98.4 (46.1)	81.5 (30.9)		−16.9	0.46

† Treatment per protocol analysis.

Reproduced with modification from reference [3].

The main mechanisms responsible for the increase in PaCO_2_ with high concentration oxygen therapy are a reduction in respiratory drive and worsening ventilation/perfusion mismatch due to release of hypoxic pulmonary vasoconstriction [2, 4–7]. Ventilation-perfusion mismatch is also a predominant gas exchange abnormality in other acute respiratory disorders such as asthma and pneumonia, with the degree of mismatch worsening with the administration of oxygen [8–14]. As a result it would be expected that high concentration oxygen therapy would cause an increase in PaCO_2_ in severe asthma and pneumonia, similar to its administration in acute exacerbations of COPD.

**Figure 1:**
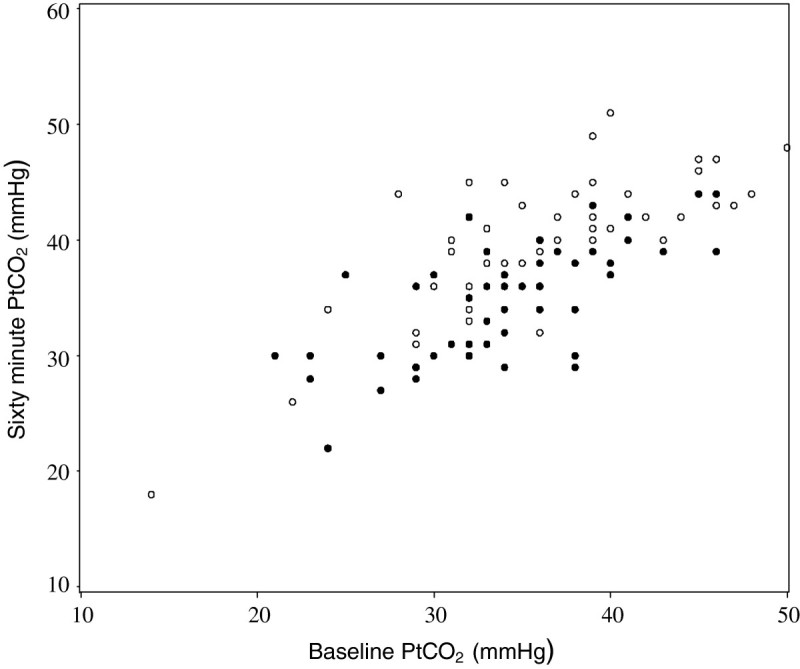
**The transcutaneous partial pressure of carbon dioxide (PtCO**_**2**_ **) levels at baseline and after 60 min of high concentration (open circles) or titrated (filled circles) oxygen in patients presenting to the Emergency Department with a severe exacerbation of asthma.** Reproduced from reference [15].

Recently a series of RCTs has demonstrated that high concentration oxygen treatment results in a significant increase in PaCO_2_ or transcutaneous carbon dioxide tension (PtCO_2_) in patients presenting with severe exacerbations of asthma [15–17]. The clinical significance of this physiological effect is suggested by the observation that 10% of patients randomised to high concentration oxygen therapy had an increase in PtCO_2_ of ≥10 mmHg and a PtCO_2_ ≥45 mmHg after 60 minutes of treatment, whereas no patients receiving titrated oxygen therapy to maintain the arterial oxygen saturations between 93 and 95% had this response (Figure 1) [15]. In a similar RCT, high concentration oxygen therapy was also shown to increase the PtCO_2_ in patients presenting with community-acquired pneumonia when compared with the titrated oxygen regimen which avoided both hypoxia and hyperoxia [18]. The three- and six-fold relative risks of an increase in PtCO_2_ of at least 4 mmHg and at least 8 mmHg respectively, suggest that this effect may potentially be of both physiological and clinical significance (Table 2).

**Table 2:** **The proportion of patients with community-acquired pneumonia with a rise in PtCO**_**2**_ **from baseline after 60 minutes of high concentration or titrated oxygen therapy**

	High concentration	Titrated	Relative risk	P value
	n (%)	n (%)	(95% CI)	
Change in PtCO_2_ ≥4 mmHg	36 (50%)	11 (14.7%)	3.4 (1.9 to 6.2)	<0.001
Change in PtCO_2_ ≥8 mmHg	11 (15.3%)	2 (2.7%)	5.7 (1.3 to 25.0)	0.007

Reproduced with modification from reference [18].

**Figure 2:**
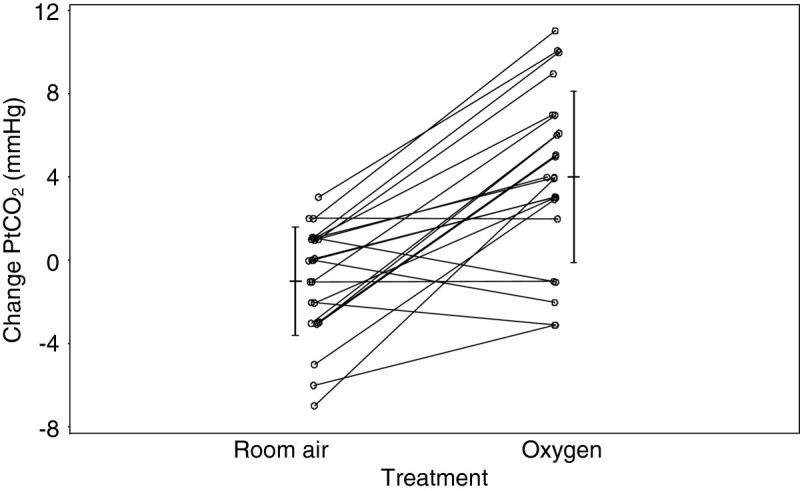
**The change in PtCO**_**2**_ **(mmHg) from baseline following breathing 100% oxygen or room air in subjects with obesity hypoventilation syndrome.** The vertical lines are the mean (central horizontal line) ±1 SD for 20 min PtCO_2_ minus baseline. Reproduced from reference [20].

Chronic respiratory failure may also occur in other chronic respiratory disorders such as obesity hypoventilation syndrome [19], so it might be expected that high concentration oxygen therapy could cause CO_2_ retention in this condition, similar to stable COPD. This physiological effect has recently been demonstrated in a randomised placebo-controlled trial of 100% oxygen and room air in patients with obesity hypoventilation syndrome and baseline hypercapnia (Figure 2) [20]. The main mechanism responsible for the worsening hypercapnia when breathing 100% oxygen was a reduction in minute ventilation, leading to alveolar hypoventilation. The clinical significance of this physiological effect was suggested by the requirement to terminate the study in one in eight of the patients studied, due to an increase in PtCO_2_ ≥10 mmHg within 20 minutes of receiving 100% oxygen therapy.

Thus there is evidence that the potential for high concentration oxygen therapy to increase PaCO_2_ is not limited to stable and acute exacerbations of COPD, but also to other acute respiratory disorders with abnormal gas exchange such as asthma and pneumonia, and chronic respiratory conditions with hypercapnia such as obesity hypoventilation syndrome. This evidence forms the basis of consensus guidelines [21] which recommend that oxygen therapy is titrated in COPD and other respiratory conditions, to ensure the maximal benefits of oxygen therapy are achieved while reducing the potential for harm due to hyperoxia.

## Authors’ original submitted files for images

Below are the links to the authors’ original submitted files for images. Authors’ original file for figure 1
Authors’ original file for figure 2
