# Supplementary figures and images for: The effect of high concentration oxygen therapy on PaCO2 in acute and chronic respiratory disorders
Source: Transl Respir Med. 2013 Apr 4;1:8. doi: 10.1186/2213-0802-1-8 (PMC6733431; doi:10.1186/2213-0802-1-8)

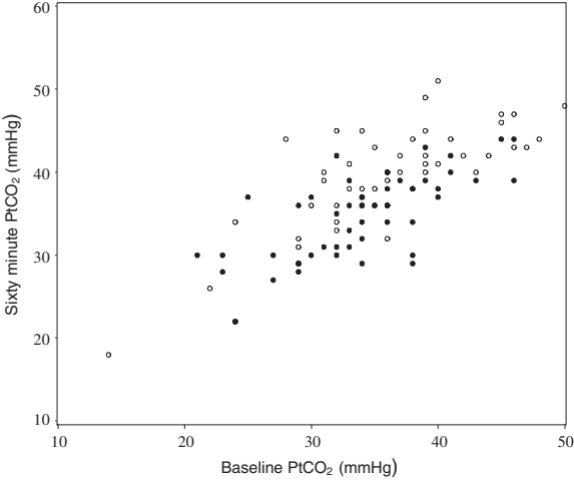

Supplement: Supplementary file 1 — Authors’ original file for figure 1 [file 40247_2013_8_MOESM1_ESM.pdf]

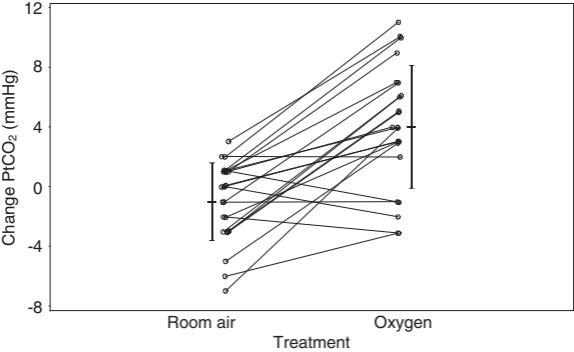

Supplement: Supplementary file 2 — Authors’ original file for figure 2 [file 40247_2013_8_MOESM2_ESM.pdf]
